# Dead Brood of *Apis mellifera* Is Removed More Effectively from Small-Cell Combs Than from Standard-Cell Combs

**DOI:** 10.3390/ani12040418

**Published:** 2022-02-10

**Authors:** Piotr Dziechciarz, Grzegorz Borsuk, Krzysztof Olszewski

**Affiliations:** Department of Apidology, Institute of Biological Basis of Animal Production, Faculty of Animal Sciences and Bioeconomy, University of Life Sciences in Lublin, 13 Akademicka St., 20-950 Lublin, Poland; piotr.dziechciarz@up.lublin.pl (P.D.); grzegorz.borsuk@up.lublin.pl (G.B.)

**Keywords:** *Apis mellifera*, hygienic behaviour, bee comb, small-cell combs

## Abstract

**Simple Summary:**

Honeybee workers are able to detect dead or infected brood in comb cells and remove it outside the nest before the infection spreads onto the colony. Such a phenomena is called an hygienic behaviour. Colonies with efficient hygienic behaviour are more resistant to diseases. Bee comb cells may vary in width. In Europe, standard-cell combs (cell width approx. 5.50 mm) and small-cell combs (cell width approx. 4.90 mm) are used. Typically, colonies are kept only on standard- or small-cell combs. We assessed the dead brood removal efficiency in colonies kept on both comb types. Simultaneous keeping of a colony on standard- and small-cell combs is a novel approach to the use of small-cell combs in beekeeping. The removal from small-cell combs was significantly more efficient than in the case of the standard-cell combs, which indicates that bees find dead brood in small-cell combs faster or devoted more attention to the removal. Better understanding of the effect of the simultaneous keeping of colonies on standard- and small-cell combs on the efficiency of hygienic behaviour may contribute to enhancement of the resistance of bee colonies to diseases.

**Abstract:**

The efficiency of the hygienic behaviour in bee colonies towards dead brood was assessed in small-cell combs (SMCombs) and in standard-cell combs (STCombs). Each colony had both types of combs in the nest on a permanent basis. Simultaneous keeping of a colony on standard- and small-cell combs is a novel approach to the use of small-cell combs in beekeeping. The number of killed pupae removed within 24 h was the measure of the hygienic behaviour efficiency. Regardless of the year, the brood in the SMCombs was uncapped and removed significantly more efficient (*p* ≤ 0.01) than in the STCombs (number of non-uncapped cells: in 2020 SMCombs = 3.79, STCombs = 11.62; in 2021 SMCombs = 2.34, STCombs = 5.28 and completely removed cells: in 2020 SMCombs = 87.46, STCombs = 80.04; in 2021 SMCombs = 96.75, STCombs = 92.66). In colonies kept simultaneously on standard- and small-cell combs, the width of the comb cells has a significant effect on the efficiency of removal of dead brood, which is removed more efficient from small-cell combs than from standard-cell combs.

## 1. Introduction

In addition to cellular and humoral immune responses against pathogens [1], social insects have developed social resistance, with nest hygiene as one of its forms [2,3]. This type of resistance was detected in termites [4], ants [5,6], stingless bees [7], and honeybees [8,9]. However, the hygienic behaviour forced by the repeated use of the same cells is specific only to honeybees [8]. Hygienic behaviour is a natural defence mechanism against brood diseases. It consists in recognition and uncapping cells with dead or infected brood and removal of the brood from the nest before the infection spreads in the colony [10,11,12]. The number or percentage of killed pupae that are completely removed per unit of time, most often within 24 h, is a measure of hygienic behaviour [13].

Hygienic behaviour can be helpful in non-chemical control of common brood diseases, e.g., chalk brood [14,15] and American foulbrood [14,16], and in reduction of *Varroa* infestations [13,17]. Long-term chemical disease control leads to immunisation of pathogens and parasites to the active substances of the applied medicaments [18,19,20,21] and to contamination of bee products [22,23].

Hygienic behaviour is modified by environmental factors, e.g., nectar flow [24], and factors of the internal environment in the nest, e.g., comb cell width [25]. As reported by Olszewski et al. [25], keeping colonies on small-cell combs (cell width of 4.93 mm) results in a significant increase in the efficiency of hygienic behaviour in comparison with colonies kept on standard-cell combs (cell width of 5.56 mm). However, it has not been clarified whether bees reared on small-cell combs are more efficient in identification of dead brood cells and removal of their contents or whether dead brood in small-cell combs stimulates worker bees to clean the cells more efficiently than brood in standard-cell combs. Summing up, the question is whether the greater intensity of hygienic behaviour in colonies kept on small-cell combs is associated with workers traits or brood traits.

The interest in small-cell combs has been aroused by reports showing that the development of *Varroa destructor* parasite populations can be reduced by rearing brood in small-cell combs instead of standard-cell combs. With its global range, the *V. destructor* mite causes large colony losses, and is therefore the biggest and most common problem of modern apiculture [26,27,28]. To date, the reduction of the development of *V. destructor* populations in brood reared in small-cell combs has been confirmed in Europe [29], Argentina [30], and Brazil [31,32]. In contrast, this has not been confirmed by studies conducted in the USA [28,33,34], New Zealand [35], and some studies carried out in Europe [36]. However, it has been shown that keeping colonies on small-cell combs exerts a significant effect on the morphological traits and the biology of worker bees. This results in a decrease in the thorax weight, head width and height, thorax width and length, width and length of fore wings, and width of the 3rd and 4th tergites [28,37,38]; additionally, it contributes to a higher effectiveness of hygienic behaviour [25] and a higher rate of springtime colony development [33] as well as extension of the lifespan of workers [39].

The aim of the study was to investigate the efficiency of dead brood removal from small-cell combs and from standard-cell combs in colonies kept simultaneously on both types of combs. This is a novel approach to the use of small-cell combs in beekeeping. Previous investigations were focused on comparison of colonies kept only on small-cell combs with colonies kept only on standard-cell combs.

## 2. Materials and Methods

### 2.1. Characteristics of Bee Colonies

**Figure 1:**
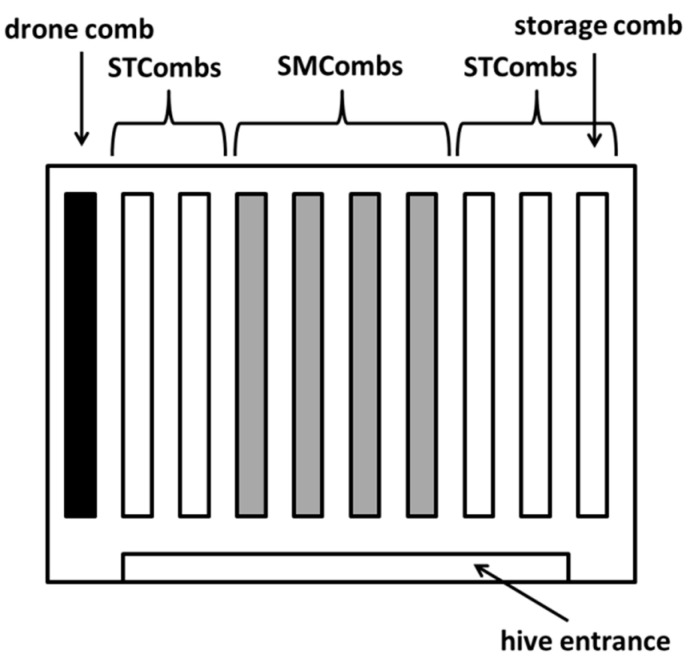
Scheme of the arrangement of the combs in the colony brood-chamber. SMCombs—small-cell combs; STCombs—standard-cell combs.

The study was conducted in the 2020 and 2021 years at the apiary of the University of Life Sciences in Lublin (51°22′ N, 22°63′ E). In each year, eight strong colonies with a similar strength and structure headed by naturally mated Buckfast sister-queens were analysed. The colonies were kept in Dadant Blatt hives. The Buckfast bee colonies kept in our apiary are very well adapted to living on small-cell combs [37], hence we used these bees in our experiment. Each colony was kept in one brood chamber with 10 frames (435mm × 300 mm) and two honey supers, each with 10 frames (435mm × 150 mm). The honey suppers were separated from the brood chamber by a queen excluder. In the centre of the nest in each colony, there were four small-cell combs (SMCombs) and five standard-cell combs (STCombs); three of the latter combs were located to the right and two to the left of the SMCombs (small-cell combs) (view facing the hive entrance) (Figure 1). Additionally, one drone-brood comb was located as the leftmost comb of the nest. The honey and beebread were stored in the standard-cell comb on the opposite side of the nest to the drone-brood comb (Figure 1). The other worker combs (SMCombs and STCombs) were almost completely occupied by brood.

### 2.2. Assessment of Hygienic Behaviour

In the second half of July, during the period without nectar flow, 100 cells with pupae were pierced in each of the colonies in one of the SMCombs and one of the STCombs (pin test). Combs with pierced brood were placed side by side to exclude the impact of the different location in the nest on the rate of dead brood removal. The pupae were pierced when their body was white and their eyes were purple (days 15–17 of preimaginal development). In each colony, the test was repeated three times in 2020 and four times in 2021. Two kinds of “Brushes” made of 100 pins (entomological pins, size No 2, diameter = 0.45 mm) were used for piercing, separate brush in the SMCombs and separate brush in the STCombs. The arrangement of the pins in the brushes facilitated piercing the pupa in the centre of each cell. The pin test was chosen, as it is a standard European method for the assessment of hygienic behaviour in selection for disease-resistant bees [13,40]. In our opinion, it is also more reliable than other methods [41]. The pierced brood was photographed 24 h after it was pricked and its removal rate was assessed by means of the digital image analysis system MultiScanBase v. 14.02 (scoring option) supplied by Computer Scanning System II, Warsaw, Poland. Non-uncapped and completely removed cells (with a completely removed pupa) were counted.

### 2.3. Measurements of Comb Cell Width

Each comb in which the brood was pierced was marked and, after young bees emerged, the cells with open brood were photographed in the centre of each comb quarter on one side of the comb. Next, in each quarter, the widths of 10 adjacent cells contacting with vertical side walls were measured [37].

### 2.4. Statistical Analysis

The results were analysed statistically using Statistica software formulas, version 13.3 (2017) for Windows, StatSoft Inc., Tulsa, OK, USA.

The data distribution was analysed with the use of the Shapiro–Wilk test. Since the data were not normally distributed, non-parametric tests were used for the analysis. The effect of the year relative to the comb type (SMCombs, STCombs) on types of pierced brood cells (non-uncapped, completely removed) was assessed using the Mann–Whitney test. The number of individual cell types between SMCombs and STCombs was compared with the pairwise Wilcoxon test.

The widths of the cells in the SMCombs (in 2020 *n* = 320, in 2021 *n* = 320) and in the STCombs (in 2020 *n* = 320, in 2021 *n* = 320) were compared with the pairwise Wilcoxon test.

## 3. Results

### 3.1. The Efficiency of Hygienic Behaviour

**Figure 2:**
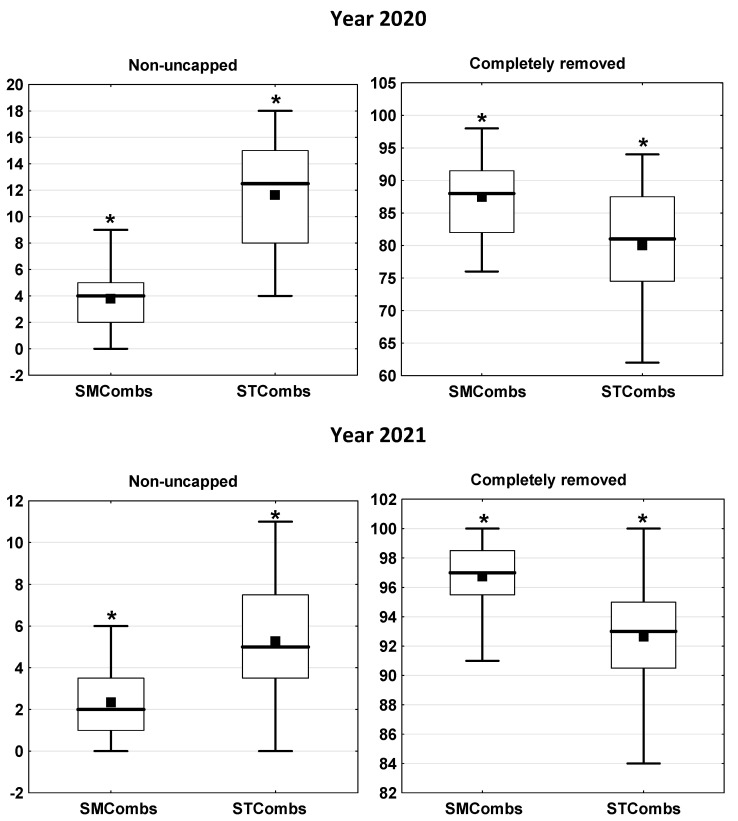
Years 2020 and 2021; number of cells with pierced brood, non-uncapped and completely removed in small-cell and standard-cell combs. SMCombs—small-cell combs; STCombs—standard-cell combs; the boxes indicate the data between the 25 and 75% quartiles including the median (black line); the black squares represent the mean; the whiskers represent the minimum and maximum values; *—the difference between SMCombs and STCombs is significant at *p* ≤ 0.01; (pairwise Wilcoxon test).

The year had a significant effect on the efficiency of hygienic behaviour. In the SMCombs and STCombs, there were larger numbers of non-uncapped cells in the 2020 than in 2021 (SMCombs—U = 234.5, *p* ≤ 0.01; STCombs—U = 88.5, *p* ≤ 0.01; Mann–Whitney test), whereas significantly higher numbers of completely removed cells were noted in the 2020 (SMCombs—U = 62.5, *p* ≤ 0.01; STCombs—U = 52.5, *p* ≤ 0.01, Mann–Whitney test). Regardless of the season, the brood in the SMCombs was uncapped and removed with significantly more efficiency than in the STCombs (number of non-uncapped cells *p* ≤ 0.01 and completely removed cells *p* ≤ 0.01) (Figure 2).

### 3.2. Comb Cell Width

The width of the SMCombs was significantly smaller (in 2020 *p* ≤ 0.01, in 2021 *p* ≤ 0.01) than that of the STCombs. The mean values of width of SMCombs in 2020 reached 4.97 mm (SD = 0.044), and in 2021 reached 4.96 mm (SD = 0.042). The mean values of width of STCombs in 2020 reached 5.57 mm (SD = 0.050), and in 2021 reached 5.56 mm (SD = 0.052).

## 4. Discussion

Simultaneous keeping of a colony on standard- and small-cell combs is a novel approach to the use of small-cell combs in beekeeping. There is no information of such a management in the literature. Previous investigations were focused on comparison of colonies kept only on small-cell combs with colonies kept only on standard-cell combs. Olszewski et al. [25] found that colonies kept on small-cell combs exhibited higher hygienic behaviour in comparison with colonies kept on standard-cell combs. In the present study, the width of the comb cells with brood in the colonies kept simultaneously on small- and standard-cell combs had a significant effect on the efficiency of dead brood removal as well. In this study, the dead brood was removed significantly more efficient from the SMCombs than from the STCombs (number of completely removed cells *p* ≤ 0.01). The more efficient removal of dead brood from the SMCombs may have been caused by the higher concentration of pheromones inside the cells, i.e., in the air filling the space between the pupa and the cell walls, which enabled workers to better identify dead brood. The higher concentration of pheromones may be related to the fact that, despite the 8–12% smaller width of the small versus standard cells, the honeybee body does not decrease in proportion to the cell width, as the thorax width decreases only by 0.8–4.4% [28,37,38]. This results in tighter filling of the cell by the pupa, and thus the presence of a smaller volume of air in the free space between the pupa and the cell walls. The fill factor, i.e., the thorax width to cell width ratio, is a measure of cell filling by the pupa [28,37,38]. At a similar level of secretion, the smaller air volume inside small-width cells is characterized by a higher degree of saturation with pheromones. It has been confirmed that odorant signals facilitate detection of dead and diseased brood in the capped cell [42,43]. In brood killed by freezing, β-ocimene and oleic acid [42] are strong inducers of hygienic behaviour, as probably in the case of pin-killed brood. Odorant signals are picked up by antennae, and hygienic workers are more sensitive than unhygienic ones [44], especially those involved in uncapping cells and initiating removal [45].

Some of the present conclusions may seem to be too bold. However, the current knowledge of the impact of keeping a colony simultaneously on standard-cell combs and small-cell combs on bee traits and colony biology is insufficient [37]. The basis for these conclusions was provided by the research conducted as part of the project “Elucidation of the phenomenon of behavioural overdominance of honeybee colonies kept on two types of combs with standard- and small-cell size,” no. 2018/31/B/NZ9/02480, financed by the National Science Centre, Poland. In colonies kept on both standard-cell combs and small-cell combs, there is an effect of the interaction of workers reared in these two types of combs, which is similar to the effect of heterosis. In terms of the colony strength, springtime colony development rate, and productivity, these colonies significantly exceed those kept only on small- or standard-cell combs [46].

Noteworthy, keeping the same colony on standard- and small-cell combs in the nest results in an increased variability of the cell width, which is similar to that in natural nests constructed without a wax foundation [30]. In such a nest, the variation in the comb cell width range from 4.17 to 8.07 mm. Worker and drone brood was reared in 4.17–6.86 mm and 5.05–8.07 mm wide cells, respectively, which indicates that both castes were reared in cells with a width in the range of 5.05–6.86 mm. We suspect that the increased cell variability in the colony results in increased variability in worker bees. The results reported by Maggi et al. [30] and our present [46] and previous [37] studies raise the question of whether the potentially differing brood rearing conditions related to the different comb cell width and the changes in the morphological traits of worker bees caste (greater variability of workers in the colony) regarded as one of the consequences of the different conditions [37] may increase the non-reproductive division of labour in the worker caste. Such a mechanism was confirmed in bumblebees, which were characterised by behavioural and physiological differences between large and small workers observed in the same colony [47]. Large bumblebee workers are more likely to work as foragers [48] and learn faster [49], whereas small ones tend to work in the nest [48] and learn more slowly [49]. On this basis, Worden at al. [49] proposed that the learning predisposition related to the body size should be considered a factor in the division of labour between large and small bumblebee workers. In the case of honeybees, the focus has so far been placed mainly on age polyethism, which probably results from the morphological similarity of worker bees, i.e., the absence of clearly distinct morphological sub-castes of worker bees. This morphological similarity is further increased by the uniformity of the comb cell width resulting from the widespread use of the wax foundation. In our opinion, it cannot be ruled out that, in colonies with a natural variability of comb cells and those reared simultaneously on standard- and small-cell combs, in addition to age polyethism, an effect is exerted by elements of morphological polyethism, which may be a compromise between specialisation and behavioural flexibility. One of the effects of this mechanism may be the greater intensification of hygienic behaviour in colonies reared simultaneously on standard- and small-cell combs.

Thus far, a subcaste of rebel workers, which are physiologically and anatomically different from normal workers, has been identified in the honeybee caste [50,51]. However, potential morphological differences of rebel workers from normal workers have not been investigated to date.

## 5. Conclusions

In colonies kept simultaneously on standard- and small-cell combs, the width of the comb cells has a significant effect on the efficiency of removal of dead brood, which is removed more efficient from small-cell combs than from standard-cell combs.

## Data Availability

The data presented in this study are available on request from the corresponding author.
